# Pilot study of a topical magnesium preparation to treat hypomagnesaemia in patients with an ileostomy

**DOI:** 10.1016/j.intf.2024.100018

**Published:** 2024-10-08

**Authors:** Jeremy Nightingale, Ibrahim Al Bakir, Franklin Adaba

**Affiliations:** a St Mark’s Hospital, Harrow, Middlesex HA1 3UJ, United Kingdom; b Chelsea & Westminster Hospital, London SW10 9NH, United Kingdom

**Keywords:** Ileostomy, Jejunostomy, Short bowel, Hypomagnesaemia, Magnesium chloride hexahydrate spray, High output stoma

## Abstract

**Introduction:**

Patients with a high output ileostomy often have hypomagnesaemia. This study aimed to determine if a magnesium chloride hexahydrate spray would maintain or increase serum magnesium in ileostomy patients with hypomagnesaemia.

**Methods:**

Outpatients with an ileostomy and having chronic hypomagnesaemia (serum magnesium <0.66 mmol/L) applied 10 sprays of magnesium chloride hexahydrate spray twice daily for 6 weeks (150 mg/day, 6.2 mmol magnesium). Serum and whole cell Mg^+2^ levels were measured at weeks 0, 1, 3 and 6. A treatment response was a rise in serum Mg^+2^ > 0.10 mmol/L at week 6, or the avoidance of a planned magnesium infusion. Serum vitamin D and aldosterone levels, and a 24-hour urinary magnesium concentration were measured.

**Results:**

6 patients completed the study, all had normal vitamin D levels (>45 nmol/L), and 5 of 6 had urinary magnesium concentrations below 0.28 mmol/L. 2 patients had a rise in serum Mg^+2^ of 0.27 and 0.13 mmol/l respectively and one avoided their planned six-weekly magnesium infusion, No patient had a fall in serum Mg^+2^ of more than 0.07 mmol/L. Serum and whole cell Mg^+2^ correlation was linear (r = 0.9181, p < 0.0001). All 6 patients complained of muscle cramping at enrolment; 5 reported an improvement or complete resolution by week 3. Serum aldosterone levels were high at the start in 4 and reduced in 3. Urine magnesium concentration increased in 2.

**Conclusions:**

A topical magnesium chloride hexahydrate spray maintains or improves serum magnesium levels in patients with an ileostomy and hypomagnesaemia and prevents muscle cramps.

**Clinical trial number:**

RD13–045

## Introduction

Magnesium is involved in many physiological processes, including enzyme activation, intracellular signalling, neuronal excitability, muscle contraction and bone formation. Problems with hydration occur in 4 % of all patients with an ileostomy [1] and many of these especially if they have a short bowel (less than 200 cm) will have hypomagnesaemia [2]. In patients with a short bowel, this is most common in those without a retained in continuity functioning colon. 41 % of those with a colon and not receiving parenteral nutrition were being given magnesium supplements or had a low serum magnesium level compared with 68 % with a jejunostomy [2].

The proposed reasons for hypomagnesaemia in patients with an ileostomy include secondary hyperaldosteronism (consequent upon sodium depletion) [3], [4], loss of absorptive sites (terminal ileum and colon), lipid in the diet (free fatty acids forming a complex with the magnesium) [5], [6] and proton pump inhibitor drugs [7].

Hypomagnesaemia alone (without hypocalcaemia or hypokalaemia) in these patients is often asymptomatic though cramps, fatigue, depression, jerky and weak muscles, ataxia, athetoid movements, cardiac arrhythmias and, if severe, convulsions have been reported [8], [9], [10], [11], [12], [13], [14]. In the long-term hypomagnesaemia will lead to a loss of bone density with a risk of fractures, and chondrocalcinosis [15].

Oral magnesium salts that have been given as a treatment for hypomagnesaemia include magnesium sulphate, chloride, hydroxide, acetate, carbonate, gluconate, lactate, citrate, aspartate, pyroglutamate, oxide and diglycinate [16], [17]. Mineral rich natural water has also been successful [18]. 1α cholecalciferol has also been used as a treatment [19], [20]. These oral treatments often do not return the serum magnesium to the normal range which may be due to unpredictable absorption. There is doubt that magnesium, like many drugs, can be adequately absorbed through the skin [21] though some experimental evidence is persuasive [22], [23]. If absorbed a topical magnesium spray may help those with magnesium depletion who do not adequately absorb magnesium preparations from their gut [23] and may offer a mode of correcting serum levels (without the need for parenteral magnesium) and helping symptoms that may be related to hypomagnesaemia.

Plasma aldosterone levels are higher in patients with an ileostomy than controls. [24] In one study 26 % of subjects with an ileostomy had an aldosterone level above the normal range and all of these had a low (<20 mmol/l) urine sodium concentration. [25].

This pilot study aimed to determine if a magnesium chloride hexahydrate spray could maintain or increase serum magnesium in patients with an ileostomy and hypomagnesaemia. It also aimed to determine if plasma aldosterone levels changed.

## Methods

Patients were recruited if they had an ileostomy formed more than 6 months previously and suffered from chronic hypomagnesaemia, defined as a serum magnesium level < 0.66 mmol/L at enrolment and either a further outpatient serum magnesium level < 0.66 mmol/L in the last 3 months, or were needing regular intravenous magnesium infusions at least once every 6 weeks for more than 18 weeks.

Exclusion criteria include severe hypomagnesaemia (< 0.25 mmol/L), diuretic use, and medication alterations (including magnesium supplements or infusions) within 4 weeks of enrolment.

At enrolment reclining/semi recumbent blood was taken for serum magnesium, whole cell magnesium, vitamin D and aldosterone, and a 24-hour urinary magnesium collection was made.

Recruits applied 10 sprays of a topical magnesium chloride hexahydrate* twice daily for 6 weeks (onto their torso and proximal limbs), delivering a topical elemental magnesium dose of 150 mg/day (6.2 mmol magnesium). The spray (0.12 ml/spray) is a liquid brine solution that is massaged into the skin and left on for at least one minute before any residue is wiped away.

Serum and whole cell magnesium levels were measured at weeks 1, 3 and 6. Serum aldosterone and a urinary magnesium concentration were measured at week 6.

A treatment response was defined as a serum magnesium level rise > 0.10 mmol/L at week 6, or the avoidance of a planned magnesium infusion during the trial without a fall in serum magnesium. While it would be most desirable for a therapy to achieve a serum magnesium within the normal range, this is rarely possible (unless intravenous magnesium is given); hence a small increase in serum level was chosen as an endpoint.

Any patient with a serum magnesium level that fell below 0.25 mmol/L or who required additional magnesium supplementation at any point during the six-week study would be deemed a trial failure and withdrawn from the study.

* : The magnesium chloride hexahydrate preparation (MagnesiumOil) was supplied by BetterYou™.

## Results

7 patients were enrolled into the study; one patient was withdrawn at week 4 due to hospitalisation for active Crohn’s disease with an intra-abdominal abscess so is not included in the patient or data analysis (see Table 1). No patient was withdrawn due to treatment failure. Four of the six patients had less than 200 cm small bowel remaining. One patient (patient 6) received 6 weekly magnesium infusions. Of those who completed the study 5 patients had a vitamin D level greater than 50 nmol/L and one had a level of 42 nmol/L. Five of 6 had baseline urinary magnesium concentrations below the minimum detection limit (<0.28 mmol/L). No patient was receiving parenteral nutrition and one had 6 weekly magnesium infusions.

**Table 1:** Patient details.

Patient number	Age (sex)	Diagnosis	Small bowel length cm	Vitamin D nmol/l
1.	61 F	Iatrogenic injury	180	82
2.	41 F	Crohn’s	160	42
3.	51 F	Crohn’s	100	68
4.	51 M	Crohn’s	110	67
5.	52 F	Crohn’s	250	133
6.	54 F	Ulcerative colitis	250	104

### Treatment outcome

**Fig. 1:**
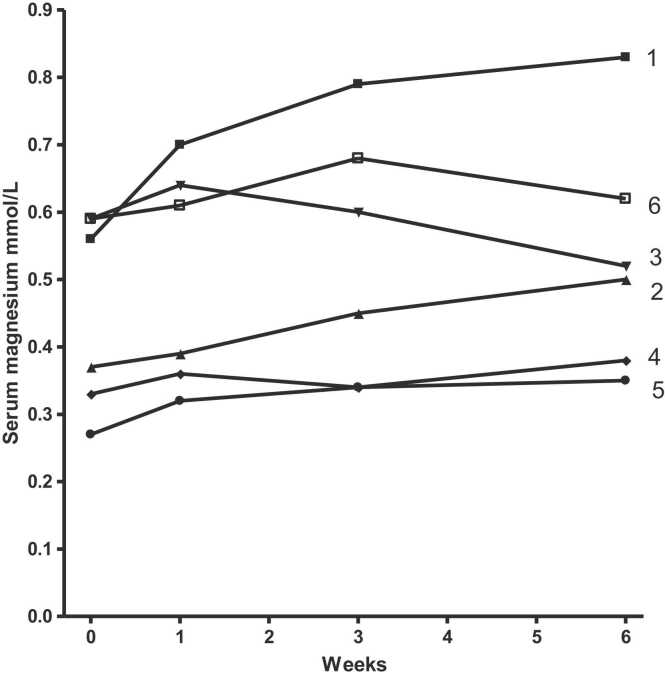
Serum magnesium after magnesium spray for 6 patients.
  No patient reported any side effects from the magnesium oil spray.
  Three patients were treated successfully. 2 patients had a serum magnesium rise of 0.27 and 0.13 mmol/L and one patient avoided their planned six-weekly magnesium infusion (maintaining a stable serum magnesium level with an increased urinary magnesium concentration). No patient had a fall in serum magnesium of greater than 0.12 mmol/L at any time point; so no patient was withdrawn on that criteria. Only one patient had a lower magnesium at 6 weeks compared to the start (0.59 mmol/L reduced to 0.52 mmol/L) and they had the shortest length of bowel (100 cm).
b) ***Urine magnesium***
  5 urine magnesium concentrations at enrolment were less than 0.28 mmol/L (the lower limit of detection) and 4 of these remained less than 0.28 mmol/L at the end of the study. 2 patients had an increase in urinary magnesium concentration.
c) ***Muscle cramps***
  All 6 patients complained of calf muscle cramping at enrolment; 5 reported significant improvement or complete resolution of cramping by week 3.
d) ***Relationship between serum and whole cell magnesium (*** Fig. 2 ***)***

a) Serum magnesium (Table 2 and Fig. 1)

**Table 2:** Magnesium results.

Patient number	Serum Mg (mmol/L)		Urine Mg 24 h (mmol/L)		Muscle cramps	Outcome*
	Start	week 6	Start	week 6		
1.	0.56	0.83	< 0.28	1.77	Resolved	Success
2.	0.27	0.50	< 0.28	< 0.28	Improvement	Success
3.	0.59	0.52	< 0.28	< 0.28	Improvement	Little change
4.	0.33	0.38	< 0.28	< 0.28	No improvement	Little change
5.	0.27	0.35	< 0.28	< 0.28	Improvement	Little change
6.	0.59	0.62	0.29	1.74	Improvement	Success*

A successful outcome for serum magnesium is considered an increase of more than 0.1 mmol/L or not needing a magnesium infusion.

* : Patient 6 did not need her 6 weekly magnesium infusion at the end of the study.

**Fig. 2:**
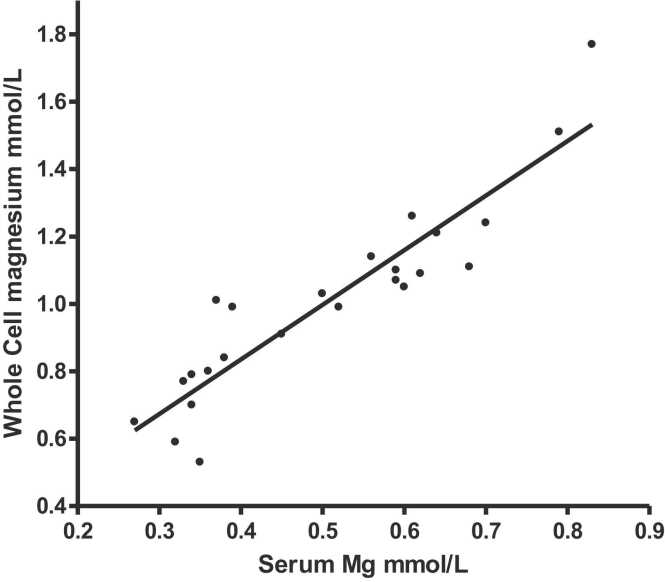
Whole cell magnesium v serum magnesium, r = 0.9181,P < 0.0001.
  Serum and whole cell magnesium correlation was strong and linear (r = 0.9181, p < 0.0001) (Fig. 2).
e) *Relationship between serum aldosterone level and serum magnesium*. (Table 3 *and* Fig. 3)

**Table 3:** Aldosterone results.

Patient	Serum aldosterone (pmol/L)*	
number	Start	Week 6
1.	2300	3250
2.	311	142
3.	5090	140
4.	149	245
5.	1510	3740
6.	8100	7240

* : Normal adult range 90-700 pmol/L

**Fig. 3:**
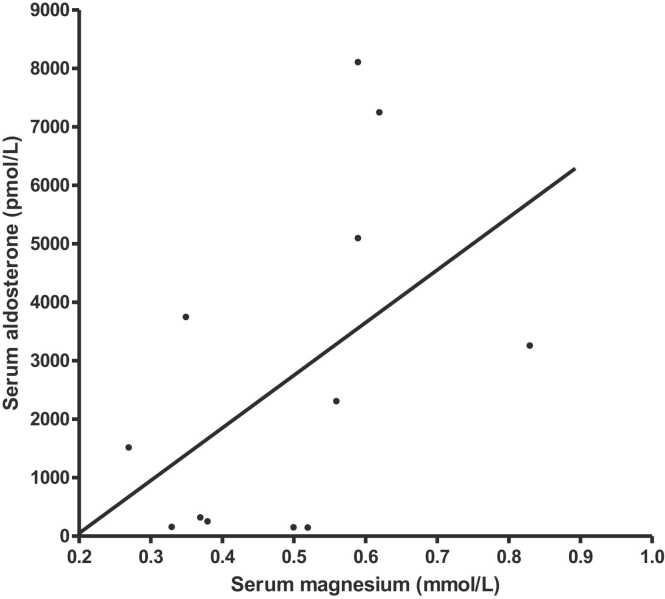
Serum aldosterone and magnesium, r = 0.502,P = 0.097.

The correlation between serum aldosterone (laboratory normal range 90–700 pmol/L) and serum magnesium was r = 0.502, p = 0.097 (Fig. 3). 4/6 had high serum aldosterone levels at the start (none had low levels) and 3/6 had a reduction and 3/6 a rise in levels at 6 weeks.

### Limitations of the study

Compliance at home can be a major problem in this patient group, so the patients studied were not asked to collect stomal output for 24 h to document that they had a high volume stomal output (>2 L/24 h when taking food and drink) though it is likely they all had one. Similarly, they were not asked to produce a 24-hour urine collection on which a random urine sodium measurement could also be made. These additional measurements may have helped with the interpretation of the serum aldosterone measurements.

We did not record all the drugs (e.g. proton pump inhibitors or vitamin D supplements) that the patients were taking during the study period. However, we did ensure that they had not changed for 4 weeks before enrollment and that they remained the same throughout the study period.

## Discussion

This is the first clinical study to examine treating hypomagnesaemia with topical magnesium chloride hexahydrate spray. 50 % (3/6) patients with an ileostomy and hypomagnesaemia were improved with this treatment, the other 3 maintained their serum magnesium levels. Muscle cramps became better in 5 of the 6 patients. This study shows that serum magnesium levels in these patients were a reliable surrogate measurement for whole cell magnesium which is commonly used as the definitive assay for magnesium status [26]. This study also shows that high aldosterone levels were common (4/6) in ileostomists with hypomagnesaemia. However, the serum aldosterone levels did not significantly change during the study.

These patients with a jejunostomy/ileostomy and hypomagnesaemia are difficult to recruit into studies as they are uncommon, often medically unstable and thus are not keen to change/try a new treatment especially if they are feeling well. In addition, the patients may also have a long distance to travel to a tertiary referral centre and may not be keen to come for repeated measurements and blood tests. The result is that these types of study always have difficult recruitment and thus small numbers.

This study does confirm that high serum aldosterone levels are common in this group of patients. High aldosterone levels are known to reduce serum magnesium levels largely by increasing renal excretion of magnesium [3], [4]. We have not shown a clear relationship between serum aldosterone and magnesium levels; however, the patient with the highest aldosterone level had the highest urinary magnesium concentration while maintaining a serum magnesium level above 0.6 mmol/l. It is likely that aldosterone is not the only determinant of the serum magnesium levels in these patients.

The consequences associated with hypomagnesaemia are well described in patients with renal or endocrine disease, alcoholism or receiving critical care but not in those with an ileostomy (and often a high output stoma) [27]. At the start of the study, 3 of the 6 patients recruited had serum magnesium measurements of less than 0.4 mmol/L, without any clinical symptoms, except cramps and/or thirst. It is common that these patients are asymptomatic unless hypocalcaemia or hypokalaemia are also present. Most clinicians will treat these low (though asymptomatic) serum levels of magnesium as there may be a risk of neuromuscular complications (e.g. fits) or cardiac arrhythmias (e.g. torsades de pointes) if untreated.

Circulating serum magnesium (as measured biochemically) is composed of magnesium in 3 states: ionised (60 %), protein bound mainly to albumin (30 %) and complexed to serum anions (10 %). As only ionised magnesium is physiologically active, it may be more relevant to measure this rather than total serum magnesium [26].

The commonly used oral treatments for hypomagnesaemia include maintaining hydration (sodium and water status), reducing lipid in the diet so that fatty acids cannot form complexes with magnesium so preventing its absorption, stopping proton pump inhibitor drugs which are only beneficial in patients with a high net secretory stomal output and needing parenteral support [28], [29]. Then drug therapy may include an oral magnesium preparation (magnesium oxide, glycerophosphate, aspartate or citrate are most commonly given) and occasionally 1α-hydroxycholecalciferol (1–9 μg daily) which increases both intestinal and renal magnesium absorption. As aldosterone levels are generally high spironolactone may have a role but due to its diuretic action it may worsen dehydration [30].

In summary this study shows that a topical magnesium chloride hexahydrate spray may be helpful in treating/maintaining the serum magnesium level in patients with an ileostomy/jejunostomy and hypomagnesaemia. This is especially the case if other oral measures have failed and before using parenteral magnesium. The spray also reduced muscle cramps.

## Funding statement

No medical staff received financial assistance for their work or time.

The magnesium chloride hexahydrate preparation (BetterYou™ MagnesiumOil) was supplied by BetterYou Ltd. www.betteryou.com. BetterYou Ltd also funded the testing and laboratory analysis on each patient throughout the duration of the trial.

## Ethical statement

Ethical approval was granted in June 2015. Informed consent was obtained, and patients consented to their data being published with the knowledge that their identity would not be disclosed. Recruitment was from 2016–2018.

## CRediT authorship contribution statement

**Jeremy Mark Darby Nightingale:** Writing – review & editing, Writing – original draft, Supervision, Project administration, Methodology, Investigation, Formal analysis, Conceptualization. **Ibrahim Al Bakir:** Data curation, Investigation, Methodology, Writing – review & editing. **Franklin Adaba:** Data curation, Investigation, Writing – review & editing.

## Declaration of Competing Interest

The authors declare the following financial interests/personal relationships which may be considered as potential competing interests: BetterYou™ paid for the investigations and provided the magnesium chloride hexahydrate preparation (MagnesiumOil). The founder and CEO of BetterYou™ was present for many of the planning stages; however, the design of the study, collating results and writing up was done by the medical team. His role was providing the funding and checking all comments that related to the composition of the product. No clinician/author received any individual financial benefit that could have appeared to influence the work reported in this paper.
